# Hybrid Plasmonic Nanostructures and Their Applications

**DOI:** 10.3390/nano12234293

**Published:** 2022-12-02

**Authors:** Sergey M. Novikov

**Affiliations:** Center for Photonics and 2D Materials, Moscow Institute of Physics and Technology (MIPT), 9 Institutsky Lane, Dolgoprudny 141700, Russia; novikov.s@mipt.ru; Tel.: +7-9032360487

The hybrid nanostructures, i.e., structures that incorporate several types of materials (for instance, metal-dielectric materials, 2D materials, etc.), constitute a dynamic and rapidly developing area of nanotechnology. One of the important directions of research in this field is the development of hybrid-plasmonic nanostructures. Controlled and reliable field enhancement based on the excitation of the plasmons in resonant metal nanostructures constitutes a prerequisite for the development of various sensing configurations. However, although many plasmonic structures have been developed, there are several problems related to their reproducibility and sensitivity that remain unsolved. In addition, such nanostructures are typically optimized in order to solve a specific problem, such as the enhancement of Raman or fluorescence signals, to cite one example. The use of hybrid materials significantly improves the characteristics of nanostructures and expands the range of possibilities for their application. This Special Issue focuses on the design, fabrication and application of hybrid-plasmonic nanostructures. It includes six scientific manuscripts, consisting of five articles and one review. An important property of hybrid nanostructures is their multifunctionality (multitasking), according to which each element of the nanostructure performs its own task. A striking example of such multifunctionality is presented in the articles investigating SERS applications [1,2]. Here, it was experimentally demonstrated that the efficiency of SERS substrates from Au films (3–9 nm), which are deposited near the percolation threshold on top of Si/Au/SiO_2_ and Si/Au/SiO_2_/graphene multilayer structures, can be enhanced seven-fold relative to Au films deposited on a standard glass substrate. Additionally, the close contact between the analyte and graphene and the nanostructured Au efficiently quenches the fluorescent background of the model analyte [1]. Thus, multifunctionality is realized, as the excitation of the gap plasmons in the multilayer structure provide additional enhancement, and the graphene layer quenches the fluorescent background. Another good example of multifunctionality is the use of the hybrid SERS substrates formed by the stabilized aggregates of Au nano-stars on a substrate coated with a layer of poly (4-vinyl pyridine) polymer, developed as a tool for visualizing the composition of living cells. The special coating of the SERS substrate provides a good cell adhesion capacity and viability, and the Au nano-stars’ long tips can penetrate the cell membrane, allowing it to receive the SERS spectra from the biomolecules inside the living cells [2]. Hybrid-plasmonic nanostructures also show great potential for biomedical applications [3,4,5]. Their multifunctionality can be exploited to develop new image-guided therapies for the treatment of cancer and other diseases. The combination of plasmonic and magnetic properties was realized in the development of core-satellite nanoparticles (NPs) consisting of Fe-based cores (30–50 nm) adorned with small Au NPs of approximately 7.5 nm. The resulting core-satellite nanocomposites obtained by the laser ablation technique are free from toxic impurities and can be used in biomedical applications, such as magnetically or photo-induced therapy and magnetic resonance imaging, amongst others [3]. Hybrid nanoparticles consisting of pH-sensitive superparamagnetic iron oxide core-gold shells (SPION@Au), chitosan (CS), and folate (FA) were developed as a doxorubicin (DOX) antitumor therapy. The synthesized SPION@Au-CS-DOX-FA spherical NPs demonstrated excellent drug loading and release capacities. These NPs show the potential for long-term anti-cancer efficacy due to their cytotoxic effect and apoptotic-inducing efficiency in SkBr3 cell lines. The results obtained in vivo reveal that the SPION@Au significantly decreased the tumor size in mice treated by magnetization. Thus, the prepared hybrid nanostructures can be used as drug formulations for the clinical treatment of breast cancer [4]. Hybrid nanostructures based on microbubbles with an air core and a shell consisting of bovine serum albumin, albumin-coated Au NPs and clinically available photodynamic dyes (zinc phthalocyanine, indocyanine green) have the potential for multimodal imaging applications in photodynamic therapy. It was demonstrated that the combination of Au NPs and photodynamic dyes influenced the fluorescent signal and probe stability. The potential of the formed probes to be used in biomedical applications was demonstrated by fluorescence tomography, scanning optoacoustic microscopy and ultrasonic response measurements taken with a medical ultrasonic device at a frequency of 33 MHz [5]. An important direction for this field of research is to improve the luminescence efficiency of certain materials that can be used widely in optical communication, biological fluorescence imaging, etc. For instance, lanthanides are of interest because of their narrow emission bandwidth, large Stokes shifts, long radiation lifetime and strong photostability. The combination of the nanocomposite systems of lanthanide material and plasmonic NPs can improve the luminescence efficiency of lanthanide materials [6]. Thus, based on the observations described above, the results of the works presented in this Special Issue can contribute to the development of research on these materials and the expansion of their biomedical applications in the future.
